# Brief motivational therapy versus enhanced usual care for alcohol use disorders in primary care in Chile: study protocol for an exploratory randomized trial

**DOI:** 10.1186/s13063-020-04589-4

**Published:** 2020-07-31

**Authors:** Nicolas A. Barticevic, Fernando Poblete, Soledad M. Zuzulich, Victoria Rodriguez, Laura Bradshaw

**Affiliations:** 1 Department of Family Medicine, School of Medicine, Pontificia Universidad Católica de Chile, Alameda 340, PO 8331150 Santiago, Chile; 2 Department of Public Health, School of Medicine, Pontificia Universidad Católica de Chile, Alameda 340, PO 8331150 Santiago, Chile; 3 Nursing School, Pontificia Universidad Católica de Chile, Alameda 340, PO 8331150 Santiago, Chile

**Keywords:** Alcohol use disorder, Brief Motivational Treatment, Intervention, Primary care

## Abstract

**Background:**

Harmful alcohol use is a leading cause of global disability and death. However, increased detection and brief intervention capacity of more severe alcohol use disorders has not been accompanied by increased availability of treatment services. Incorporating treatment for such disorders into primary care is of paramount importance for improving access and health outcomes. This study aims to estimate the effectiveness of a Brief Motivational Treatment (BMT) applied in primary care for treatment of these disorders.

**Methods:**

A parallel-group, single-blinded, severity-stratified, randomized clinical trial will test the superiority of BMT over enhanced usual care. Eligible participants will be those seeking treatment and who fulfill DSM-V criteria for alcohol use disorder and criteria for harmful alcohol use. With an estimated a loss to follow-up of 20%, a total of 182 participants will be recruited and equally randomized to each treatment group. The intervention group will receive an adaptation of the motivational enhancement therapy, as manualized in Project MATCH. This treatment consists of four 45-min sessions provided by a general psychologist with at least 3 years of primary care experience. The primary outcome is the change from baseline in the drinks per drinking day during the last 90 days, which will be captured using the Timeline Follow Back method. Secondary outcomes will describe the changes in alcohol use pattern, motivational status, and severity of the disorder.

All participants will be analyzed according to the group they were allocated, regardless of the treatment actually received. Mean differences (MD) will be computed for continuous outcomes and relative risks (RR) and RR reductions (RRR) for dichotomous results. Linear models will deliver the subgroup analyses. Missingness is assumed to be associated with the baseline alcohol use pattern and severity, so a multiple imputation method will be used to handle missing data.

**Discussion:**

This trial aims to test the superiority of BMT over enhanced usual care with a reasonable superiority margin, over which the BMT could be further considered for incorporation into PC in Chile. Its pragmatic approach ultimately aims to inform policymakers about the benefit of including a brief psychosocial treatment into PC.

**Trial registration:**

ClinicalTrials.gov NCT04345302. Registered on 28 April 2020

## Administrative information

Note: the numbers in curly brackets in this protocol refer to SPIRIT checklist item numbers. The order of the items has been modified to group similar items (see http://www.equator-network.org/reporting-guidelines/spirit-2013-statement-defining-standard-protocol-items-for-clinical-trials/).

**Table:** 

Title {1}	Brief motivational therapy versus enhanced usual care for clients with alcohol use disorder in primary care in Chile: study protocol for a pilot randomized trial
Trial registration {2a and 2b}.	ClinicalTrials.gov, NCT04345302, Registered 28 April 2020, https://clinicaltrials.gov/show/NCT04345302
Protocol version {3}	Issue date: 10 April 2020 Protocol version number: 1
Funding {4}	The Chilean ‘*Fondo Nacional de Desarrollo Científico y Tecnológico*’ will fund this study through a research grant (FONDECYT 11190874). The Pontificia Universidad Católica de Chile will provide administrative support.
Author details {5a}	NB conceived the study. NB and FP developed the first version of the protocol. All authors contributed to refinement of the study protocol and approved the final manuscript.
Name and contact information for the trial sponsor {5b}	Trial sponsor-investigator: NB Address: Diagonal Paraguay 362, Santiago - Chile. Faculty of Medicine, Pontificia Universidad Católica de Chile.
Role of sponsor {5c}	The funding agency (FONDECYT) oversees the whole progress of the project through periodic administrative and scientific reports, but has no role in the design, execution, and analysis of this study.

## Introduction

### Background and rationale {6a}

Alcohol consumption continues to have a massive negative impact on health. During 2016, the harmful use of alcohol accounted for 5.3% of all deaths and 5.1% of all disability-adjusted life years (DALYs) worldwide, representing a major preventable risk factor for disability and death [1]. Alcohol consumption is associated with digestive diseases, cancer, diabetes mellitus, epilepsy, and cardiovascular diseases, among other health problems. Among the 230 diseases and health problems to which alcohol is related, alcohol use disorders (AUDs) account for 14% of all alcohol-attributable DALYs, preceded only by injuries and digestive diseases [1]. Despite the existence of effective treatment options [2–4], there remains a significant treatment gap between the number of individuals affected by AUDs and those who receive treatment. This is probably the most substantial gap among all mental health disorders [5]. In Chile, less than 5% of people with AUDs received an appropriate intervention during 2014 [6].

The programs for screening, brief intervention, and referral to treatment (SBIRT) attempt to bridge this gap and have resulted in increased detection and provision of brief interventions for risky alcohol usage [7]. However, brief interventions are insufficient for more severe cases (i.e., alcohol dependence) [8]. In Chile, only 11% of people with alcohol dependence received treatment 6 months after the referral from the SBIRT program. A sensible approach to tackle this issue would be to advance the incorporation of services for AUDs in primary care (PC). This strategy could remove several access barriers and make use of the client’s motivational momentum. Additionally, a treatment for alcohol dependence within PC would provide care in a less stigmatizing way. Moreover, such a treatment could contribute to healthcare integration since alcohol use is intimately associated with relevant health outcomes [9].

Evidence of effective options to treat alcohol dependence in PC is still scarce but starting to grow. Finn et al. [10] studied a structured treatment in PC (i.e., the 15-method) in comparison to specialized treatment in secondary care. A 15-min intervention delivered by physicians in primary care behaved similarly to specialist treatment for moderate severity alcohol use disorder, even when clients in PC attended an average of 2.9 visits in contrast to clients in specialist care attending an average of 4.7 sessions. Brown et al. [11] reported another approach to treatment in PC consisting of a telephone and mail adaptation for a psychosocial intervention. They showed mild effects on male participants; however, we know little about the severity of the alcohol use problem, other than half of the participants met diagnostic criteria for dependence and the other half met the criteria for abuse. Finally, Nadkarni et al. [12] studied a four-session treatment for men with harmful alcohol use. In this study, lay counselors delivered the intervention, showing remission of harmful alcohol use in 36% of the participants.

All of these interventions show significant benefits from well-designed treatments that suit the clients’ needs and are coherent with the functioning of PC. Notably, a brief therapy in PC could be the natural continuation of SBIRT programs and provide high quality of care for a variety of clients. The motivational enhancement therapy (MET) [13] is of particular interest to PC because it is short (i.e., four sessions), its efficacy is supported by robust evidence [13, 14], and it is based on the motivational interview (MI) [15], which is a prominent counseling style in PC. In fact, several of MET’s techniques, like personalized feedback and person-centered counseling style, are present in the PC treatment modalities mentioned above.

Even though the effectiveness of MET has been established in well-designed clinical trials, their results cannot be directly extrapolated to PC. Mainly, the larger clinical trials on MET effectiveness (i.e., project MATCH [13] and the United Kingdom Alcohol Treatment Trial [UKATT] [14]) compare it with other active treatments, where specialized counselors provide MET and the active comparator. On these trials, MET showed an effectiveness similar to the other more extensive treatments. However, we do not know if this effectiveness will remain when applied by a general psychologist. In fact, the provider’s professional credentials modify the effect of motivational treatments, with a higher qualification associated with better outcomes [16]. On the other hand, clients with AUDs have access to a variety of unspecialized services in PC; it could be the case in this setting that usual care is adequate for a large number of clients with a mild form of dependence. For instance, a client could have a consultation with a psychologist, access several social services through a social worker, have an appointment with a physician and get a prescription for psychoactive drugs and disulfiram, or be referred to secondary care. In other words, as MET has not been compared with usual care in PC, and as some clients recover from AUDs spontaneously in the community, we cannot anticipate the net effect of MET in this setting. Altogether, the therapy cannot be introduced to Chilean PC without adaptation and local evidence that confirms the effect.

This trial aims to study the effectiveness of a Brief Motivational Treatment (BMT), which is an adaptation of MET’s procedures to Chilean PC, compared to enhanced usual care (EUC).

### Objectives {7}

This exploratory trial aims to estimate the effectiveness of a BMT for AUD provided in PC. The underlying question is whether Chilean PC should incorporate this treatment among its regular programs based on its effectiveness.

To answer this question, we will undertake a randomized comparison between the manualized BMT and EUC. Our main hypothesis is a superiority one:

- Participants under BMT will perform better than EUC in the reduction of alcohol consumption.

Also, there are ancillary questions that deserve special attention. The following hypotheses will help with the explanation of the results:

- ◦ Active BMT components (i.e., the working alliance and fidelity to the MI strategies) mediate the effect.
- ◦ Participant’s AUD severity mediates the effect.
- ◦ Participants under BMT will receive a higher amount of additional care (physician consultations, social worker consultations, participation in alcoholic anonymous, and others).

### Trial design {8}

The study is a parallel-group, single-blinded, randomized clinical trial to test the superiority of BMT over EUC. The allocation ratio is 1:1. The study is also pragmatic: the participants should be representative of PC clients and the study therapists will have a competency profile similar to a regular psychologist in PC [17].

## Methods: participants, interventions, and outcomes

### Study setting {9}

The trial will take place in PC centers in Santiago, Chile.

### Eligibility criteria {10}

#### Participants

The protocol captures typical PC clients to maximize external validity. Hence, participants will be recruited from the SBIRT program currently conducted in PC centers, as well as from physician referrals. Our inclusion criteria follow previous robust clinical trials to enhance comparability (the project MATCH [13] and the UKATT study [14]).

Any client with an Alcohol Use Disorders Identification Test (AUDIT) > 15 will be considered for eligibility. We will include clients who fulfill criteria for alcohol use disorder according to the Diagnostic and Statistical Manual of Mental Disorders V [18] (DSM-V) and criteria for harmful alcohol use during the last month, i.e., five or more heavy drinking occasions in the last month (5 or more drinks in men, 4 or more in women), or an average use of 14 or more drinks a week in men, and 7 or more in women. Also, alcohol use should be the main problem motivating participants to seek treatment. In case of doubts about the eligibility of a potential participant, the criterion to follow is whether this individual would be offered treatment in PC under normal conditions.

Additionally, a clinician will verify the need to refer the participant to a detoxification service before enrolment into the trial, as well as referrals to address any pressing issue (i.e., homelessness, unstable medical pathology, developing legal situation, and so forth).

The participation of a significant other that can help the client through the treatment will be encouraged from the beginning.

#### Exclusion criteria

- *Clients under 20 years old*: In Chile, younger clients attend specific providers under a specific administrative regimen (the Explicit Health Guarantees system). Therefore, regulatory restrictions preclude the recruitment of participants under age 20.
- *Clients in whom alcohol use is not the main problem*: Following the methodology of the UKATT study [14], clients with a problematic consumption of other substances (e.g., marijuana, cocaine) may participate in the trial, provided that alcohol is currently their primary source of problems. Given that the UKATT study demonstrated effectiveness in people with more than one substance use disorder, we will follow that approach to include clients as they exist in the real world.
- *Clients who leave the area or are unable for follow-up contact*: Participants who will leave the area before completing the 6 months of follow-up or are unable to provide two alternative contacts.
- *Clients with severe mental comorbidity*: Clients with psychotic disorders, schizophrenia, active suicidal ideation, who currently represent a risk of aggression towards themselves or towards third parties, impulse control disorders, or who have any other severe mental disorder. In this regard, we are following a restrictive exclusion criterion, similar to that of the MATCH project [13]. Additionally, these clients are not treated in PC.
- *Clients with severe cognitive impairment, illiteracy, or unable to follow treatment in Spanish*.
- *Clients who are concurrently receiving or planning to receive other psychosocial treatment for AUD other than EUC*, i.e., formal professional treatment outside of PC. Participation in community services and Alcoholics Anonymous is permissible.
- *Clients who have previously participated in the study* or whose family members are or have been participants.

### Who will take informed consent? {26a}

A certified clinician in the role of research assistant (RA) will take informed consent after confirming participant eligibility. The informed consent will be taken in person, at each clinical site.

### Additional consent provisions for collection and use of participant data and biological specimens {26b}

Participants will be asked for consent to use their anonymized data in scientific publications. No biological specimens will be collected in this trial.

## Interventions

### Explanation for the choice of comparators {6b}

To study the effect of the BMT in PC, we will compare it with usual care as it occurs in Chilean PC. This comparator is relevant since it acknowledges the pragmatic goal of this trial: whether Chilean PC should incorporate this treatment among its regular programs based on its effectiveness.

Although this comparator is appropriate for the research question, its usage raises some problems. The main issue we foresee is a high variability of the services that constitute usual care between different clients and across different PC centers. The services could range from a delay in treatment due to a lengthy waiting list, to multiple timely interventions provided by an organized psychosocial team. In an extreme scenario, if this heterogeneity is too high, it could complicate the statistical analysis.

However, in the real-world scenario where the study will take place, we think it is reasonable to compare BMT against EUC. Based on local data, we assume that over 80% of AUD cases will be waiting for a consultation in the specialized services and, in the meantime, receiving a low level of non-specific services in PC. Thus, these cases compose a homogeneous cohort where we expect the typical evolution of AUD within PC. It is still possible that a proportion of these clients will receive some treatment modality (not BMT) or present spontaneous remission, so we are taking this fact into account for sample size calculation.

### Intervention description {11a}

#### BMT

Participants in the intervention group will receive the BMT, which is a PC adaptation of the MET as manualized in the Project MATCH [19]. This treatment consists of four 45-min sessions, provided by a psychologist at weeks 1, 2, 6, and 12. The first three sessions, occurring during the first 6 weeks, are more active regarding the behavioral change, while the last session functions as closure and review of the process. If a participant asks for more support, they will be able to attend up to two extra sessions before the last one.

The main adaptations to the original Project MET manual are:

- The translation into Chilean Spanish.
- An update of MI concepts used throughout the manual using the last edition of the MI reference book.
- The addition of companion training material that includes a demonstrative video and practical exercises on the MI strategies.
- A personalized feedback procedure based on the evaluation used in the trial and the local epidemiological situation.
- The addition of information on additional resources available in the PC center and the community to be included in the “menu of options.”
- The addition of a module regarding the coordination of health services in the Chilean PC context. This supplementary material is meant to facilitate the participants’ access to services complementary to BMT within the PC center (i.e., social services, medical services, among others).

#### Selection and supervision of the providers

We will recruit general psychologists or other psychosocial professionals with at least 3 years of experience in PC. They will receive training in BMT and then will demonstrate proficiency in a simulated client session (or will provide a taped interview showing a proficient application of BMT).

During recruitment, therapy sessions will be recorded, and 10% of them will be reviewed using an MI proficiency scale. Then, a feedback report on the fidelity to MI will be issued and discussed with each therapist. The instrument to be used will be chosen during the pilot among the available validated tools [20].

#### Enhancement of usual care

All participants will receive an educational brochure on alcohol use disorder, with self-help materials and guides on how to get additional support.

The physicians within the PC center will also receive information on how to diagnose AUD, prescription guides for the medications that are available for treating these disorders in the PC center (mainly disulfiram and any other if available), and directions on when and where to refer clients for treatment.

### Criteria for discontinuing or modifying allocated interventions {11b}

As the BMT will be added to EUC, all participants will be receiving the current standard of care in Chile. Also, psychosocial treatments based on MI are not expected to induce severe adverse events.

### Strategies to improve adherence to interventions {11c}

Before each session, the participants will receive a confirmation call. They will additionally receive public transportation vouchers for their attendance to assessments.

### Relevant concomitant care permitted or prohibited during the trial {11d}

During the trial, participants will continue to receive regular medical and social care at their health center. These services may include prescriptions for mental health issues and alcohol (disulfiram, anti-craving, anti-depressants, and other medications), social assistance, and the full spectrum of primary health care. Nevertheless, they will not receive other psychosocial interventions for AUD in the health center.

Additionally, participants are asked not to initiate other formal psychosocial treatment for alcohol use disorder until trial termination. This restriction does not include non-professional community services, such as attendance to Alcoholics Anonymous or other informal support.

The study record will keep track of the days when a participant receives additional treatment (if any). Even though it is prohibited, a participant that eventually receives additional formal psychosocial treatment for AUD will continue to receive the allocated treatment and will complete follow-up, and their data will be included to be analyzed according to the original allocation group.

### Provisions for post-trial care {30}

Once the trial has finished, the participants will continue their usual health care at their PC centers. There will not be special post-trial care.

### Outcomes {12}

#### Primary outcome

The primary outcome is the change from baseline in the drinks per drinking day (DDD) during the last 90 days (i.e., the previous 12 weeks), measured at 6 months follow-up. The DDD will be aggregated using means.

The DDD constitutes a relevant outcome because the frequency and quantity in which alcohol is consumed mediate the occurrence of behavioral disorders and health deterioration in clients with AUD. Even though a more comprehensive result, such as clinical recovery or improved quality of life, would be of more clinical significance, these outcomes exceed a short-term, exploratory trial. We have chosen DDD because it has shown to be sensitive to treatment and because it allows comparability with influential clinical trials [21].

#### Secondary outcomes

- The number of participants with a low-risk alcohol use pattern estimated by the number of days of consumption, of abstinence, and intoxication during the last 90 days, aggregated using the proportion of participants per group, at 6 months follow-up. Additionally, the most extended period of abstinence since enrolment will be estimated at 6 months follow-up. The number of abstinence days of each participant will be aggregated using means.
- The change from baseline in the negative secondary consequences of alcohol consumption will be measured using the DrInC-2R questionnaire [22] measured at 6 months follow-up and aggregated using means.
- The change from baseline in the severity of the AUD using the Substance Dependence Severity Scale (SDSS) at 6 months follow-up [23]. Means will be used to aggregate participants’ DAYS, SEV, and WORST SEV scores for alcohol. The severity of AUD is a critical outcome in two ways: first, we hypothesize that the severity should decrease with treatment; second, severity is expected to predict treatment effect, so it could help identify a specific range of severity in which BMT is more effective.
- The change from baseline in the motivational stage measured with the SOCRATES scale [22] at 6 months follow-up. The proportion of participants that improve their motivational stage will be used to aggregate the measurement in each group. This scale was designed to classify clients in one of the stages of change according to Prochaska and DiClemente [24] and is expected to mediate client recovery and predict treatment effect.

#### Other instruments

The therapeutic alliance will be measured after each treatment session (four times during the trial) using the Working Alliance Inventory short form in its client and therapist versions (WAI-C and WAI-T) [25].

Finally, we will collect baseline demographic variables (age, sex, years of education, employment status, and marital status).

### Participant timeline {13}

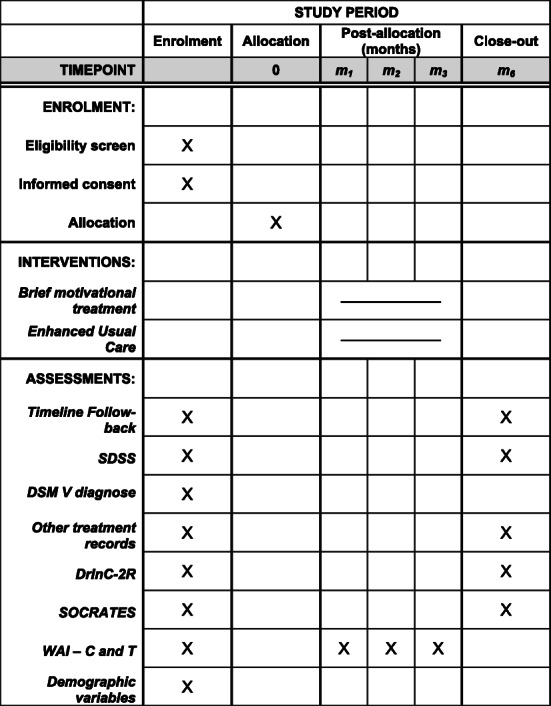



### Sample size {14}

Given that there is a spontaneous reduction of alcohol consumption in control groups of 14% [26], and that the reduction attributed to psychosocial treatments in the literature is around 40% [27], we set a superiority margin δ of 26% reduction in alcohol consumption. The following two hypotheses correspond to the superiority test:

- H_0_: DDD_EUC_ − DDD_BMT_ = < δ;
- H_a_: DDD_EUC_ − DDD_BMT_ > δ.

We used local data and the results of project MATCH as references for sample size calculation. Project MATCH [13] recruited participants with AUD in the outpatient setting and observed a standard deviation (SD) of 6.29 drinks, which is similar to reports in Chilean PC [28]. On admission, participants in the MATCH study consumed 11.5 DDD, which should be similar in our population. We expect a decrease of 14% in the EUC group [26], that is, to 9.89 DDD, and a reduction to 40% in the intervention group [27, 29], that is, a decrease to 6.9 DDD.

The sample size was obtained using the formula for means comparison in superiority studies of parallel groups, according to the methodology described by Chow [30]. The computation was made using the R statistical software [31] with the package provided by the same author [30, 32]. We obtained a sample size of *n*_1_ = *n*_2_ = 76 using the following parameters: *α* = 0.05; *β* = 0.2 : *σ* = 6.29; *δ* = 2.99; *κ* = 1 (where *α* is the type 1 error, [1- *β*] the power, *σ* the standard deviation, *δ* the expected difference between both groups, and *κ* the allocation ratio between both groups).

Considering an expected loss to follow-up of 20%, we will have to recruit 91 participants for each group, which is 182 participants in total.

### Recruitment {15}

The SBIRT program will be the primary recruitment source. Annually, this program detects roughly 60 people looking for treatment for AUD in each PC center. Working in close coordination with the local SBIRT program administrator, an RA will contact these individuals and invite them to participate in the study. Also, the clinical staff in the center will be able to refer potential participants to the study.

In case participant enrolment is slow, we will advertise the study in the community.

## Assignment of interventions: allocation

### Sequence generation {16a}

Participants will be randomly assigned to either control or experimental group with 1:1 allocation as per a computer-generated randomization schedule stratified by site and the baseline SDSS-DAYS score using permuted blocks of random sizes. The cut point for the severity score will be determined during the trial pilot. To ensure concealment, we specified the block sizes in a separate document with restricted access.

### Concealment mechanism {16b}

An online, central randomization service will randomize the participants to either group. This service assures concealment since it only reveals the sequence after the completion of baseline measurements and the confirmation of eligibility.

### Implementation {16c}

During the intake visit, an RA will first administer the baseline measurements, then check the inclusion/exclusion criteria, and finally, ask the central randomization service to assign the participant. The RA will use an online platform to complete the assessments and the randomization.

## Assignment of interventions: blinding

### Who will be blinded {17a}

An RA blinded to the allocation of the participant will perform the follow-up assessments. Due to the nature of the intervention, the participants and the therapists will know in which group they are participating; however, they will be strongly instructed to not disclose their allocation at the follow-up assessments. At trial closure, a statistician blinded to allocation will conduct the data analysis.

### Procedure for unblinding if needed {17b}

Not applicable since both participants and therapists are not blinded, and any undesired effects of the intervention will be managed without the need for unblinding.

## Data collection and management

### Plans for assessment and collection of outcomes {18a}

#### Primary outcome

The DDD during the last 90 days will be obtained using the Timeline Follow Back [33] (TLFB) procedure. The TLFB uses a calendar with key dates as memory aids to enhance recall. This method will also record other treatments that the participants received and capture the alcohol use pattern, which is defined as the typical number of drinks consumed in a drinking occasion in the previous 90 days, the frequency of heavy drinking days during the last 90 days (> 4 standard drinks in women and > 5 in men), and the total number of standard drinks consumed in a week during the last 90 days. The reliability of these measurements using TLFB lies between 0.7 and 0.9 [33].

#### Secondary outcomes

##### Substance Dependence Severity Scale (SDSS) (Spanish version) [23]

This instrument measures the severity of the dependence linked to the DSM-V diagnostic criteria by assessing both the frequency and severity of symptoms during the last 30 days. The DAYS score varies on an 8-point scale ranging from 0 (symptom did not occur) to 7 (symptom occurred every day of past 30). The SEV and WORST SEV severity variables are scored on a 6-point scale ranging from 0 (absent) to 5 (extreme), with a score of “2” indicating that the diagnostic criterion has been met. Lower scales scores represent less severe dependence, and higher scale scores reflect more severe dependence. This scale is reliable and sensitive to change in the context of addiction treatments. Additionally, the scale has a good correlation with other severity measurements, such as the Addiction Severity Index (ASI), the Drinker Inventory of Consequences (DrInC), and the Global Assessment Scale [34]. Its test-retest reliability for alcohol is excellent: ICC 0.90 [0.79–0.88] for severity and ICC 0.82 [0.77–0.86]. The reliability and concurrent validity of the Spanish version have also been studied and behave very similarly to the original scale [35]. The RAs will be trained on the procedures to apply this instrument according to what has been reported by the authors.

##### Drinker Inventory of Consequences (DrInC-2R)

This inventory addresses the negative consequences of alcohol abuse during the last 3 months using five sub-scales: physical, interpersonal, intrapersonal, social responsibility, and impulse control. Its global score ranges from 0 to 150, with higher scores indicating higher consequences. The test-retest reliability for total consequences is 0.88 (ICC), and the sub-scales range from 0.69 to 0.92 (ICC) [36]. Even though the reduction of repercussions is at the heart of treatment objectives, we did not choose it as the primary outcome because it is harder to measure and less sensitive in the short-term. This inventory will be completed by the participant with the supervision and assistance of the RAs.

##### SOCRATES

This 19-item scale measures the motivation in a continuum of three main sub-scales [taking steps (score range 8–40), recognition (7–35), and ambivalence (4–20)] that explain 45% of the variance and have good internal consistency (Cronbach’s alphas between 0.87 and 0.96), with excellent test-retest properties (interclass correlations ranged from 0.82 to 0.94) [37]. This scale will be completed by the participant with the supervision and assistance of the RAs.

##### Outcome adjudicators

In this study, the RAs will be a small group of health professionals (i.e., nurses, psychologists, or social workers) who have experience in mental health in PC and who will complete a training program to standardize data collection procedures and ensure the skillful application of the psychometric instruments according to the test manuals.

### Plans to promote participant retention and complete follow-up {18b}

To minimize participant’s burden in the process of data collection, they will receive economic compensation for attending the two follow-up sessions. Furthermore, to reduce attrition and maximize completeness of data, we have put special care in keeping the data to the minimum necessary for the trial objectives.

To encourage attendance to therapy and follow-up sessions, a centralized appointment and reminder system will contact the participants in advance.

Despite these strategies, it is anticipated that some participants will not adhere to the follow-up schedule, the therapeutic scheme, or other crucial aspects of the protocol; however, we will include all participants in the analysis to be consistent with an intention-to-treat approach.

### Data management {19}

In this trial, all data will be entered electronically at the site where data originated using a tablet computer. Customary verification algorithms will ensure completeness and validity at the moment of data entry. There will not be any paper copies of the psychometric instruments or the demographic data forms other than what is required for the treatment sessions (i.e., structured feedback). These procedures avoid data transcription and ensure the integrity from the moment of collection. The database will be stored in a private server and will be backed-up weekly.

### Confidentiality {27}

Data will be anonymized to ensure confidentiality through a unique participant number that will link the electronic data with the participant’s identification. The informed consent document will be the only record containing both the identification number and the participant’s personal information. This document will be stored in a secure place at each site and transferred monthly to the central coordinating center, where it will be stored for 5 years after study closure. A formal presentation to the Ethics committee is necessary to access these documents.

### Plans for collection, laboratory evaluation, and storage of biological specimens for genetic or molecular analysis in this trial/future use {33}

Not applicable as no biological specimens will be collected as part of this trial.

## Statistical methods

### Statistical methods for primary and secondary outcomes {20a}

We will compare the BMT group with the EUC group for every main outcome listed in Table 1. *T* tests will compare continuous results, and chi-squared tests will compare dichotomous outcomes. We will compute mean differences (MD) for continuous outcomes, and relative risks (RR) and RR reductions (RRR) for dichotomous results, with their corresponding 95% confidence interval. *P* values will be reported to four decimal places, with *P* values less than 0.001 reported as < 0.001. The latest version of R [31] will be used to perform the statistics. Table 1 summarizes the methods for the analysis of each outcome.

**Table 1:** Statistical methods for outcomes and ancillary analyses

Outcome	Hypothesis	Outcome measure	Methods of analysis
**1) Primary**			
a) Drinks per drinking day at 6 months	BMT reduced outcome from baseline to 6 months	Drinks per drinking day during the last 90 days in the Timeline Follow Back [continuous]	*T* test
**2) Secondary**			
a) Alcohol use pattern at 6 months	Back to a low-risk use or abstinence after the treatment	Presence of a low-risk pattern: less than 100 g of ethanol a week and no binge drinking occasions (i.e., more than three SD in women and 4 in men) during the last 90 days in the Timeline Follow Back [binary]	Chi-squared test
b) Frequency of heavy drinking at 6 months	Reduction	Number of heavy drinking occasions (i.e., more than three SD in women and more than four in men) during the last 90 days in the Timeline Follow Back [continuous]	*T* test
c) Most extended period of abstinence during the last 3 months	Augmentation	Number of days of abstinence within the last 90 days in the Timeline Follow Back [continuous]	*T* test
d) Severity of dependency at 6 months	Reduction	Score in the Alcohol DAYS, SEV, and WORST SEV score in the Substance Dependence Severity Scale (last 30 days) [continuous]	*T* test
e) Alcohol related negative consequences	Reduction	Total consequences score in the Drinker Inventory of Consequences [continuous]	*T* test
**3) Subgroup analyses**			
a) High v/s low severity	Greater effect in low severity.		
b) Motivational level	Higher motivation intensifies the treatment effect.		
c) Educational level	Higher education intensifies the treatment effect.		
d) Male v/s female	Sex interacts with treatment effect.		
**4) Sensitivity analyses**			
a) Per-protocol analysis			a) *T* test/chi-squared
b) Adjustment for baseline variables			b) Linear model (multivariate regression)
c) Missing data imputation			c) Multiple imputation (missing-at-random assumption)

### Interim analyses {21b}

Not applicable. We do not expect that this intervention added to usual care increases the rate of adverse events or complications. On the other hand, the recruitment rate and sample size make interim analyses impractical.

### Methods for additional analyses (e.g., subgroup analyses) {20b}

Linear models with the appropriate interaction factors will deliver the subgroup analyses. We will examine the residuals to check for model assumptions and common problems with the errors, the predictors, and the model structure. If necessary, robust regression methods or transformations will be used.

### Methods in analysis to handle protocol non-adherence and any statistical methods to handle missing data {20c}

For the analysis of missing data, we will follow the missing-at-random assumption, which allows valid analysis independently of the missing value mechanism. We assume, therefore, that missingness is associated with the baseline alcohol use pattern after conditioning on covariates, which is a reasonable assumption since higher alcohol usage leads to treatment failure and reduced follow-up. In this scenario, we will use a multiple imputation method to handle missing data.

On the other hand, to prevent attrition bias, outcome data from all randomized participants will be analyzed according to the group they were allocated, regardless of protocol adherence.

### Plans to give access to the full protocol, participant level-data, and statistical code {31c}

The full protocol, dataset, and statistical code are available in the Open Science Foundation repository, DOI 10.17605/OSF.IO/6BA3W

## Oversight and monitoring

### Composition of the coordinating center and trial steering committee {5d}

This trial will be conducted and monitored by a small research team made up of the authors and the support staff. This team includes a person with data management training who will oversee data collection and integrity and a general coordinator to handle daily operations at the sites.

### Composition of the data monitoring committee, its role and reporting structure {21a}

Not applicable. This trial is of relatively short duration and presents minimal risks for participants since BMT is not known to have adverse effects on clients. Any typical emergency situations will be managed as usual in the health care context. For these reasons, it seems unnecessary to have a formal data-monitoring committee (i.e., a multidisciplinary group of people independent from the trial researchers that decide on trial modification or suspension).

### Adverse event reporting and harms {22}

The brief motivational therapy is not supposed to increase health risks in clients; however, due to the behavioral nature of the alcohol use disorder, it is expected that some common problems could arise in the participants. High-risk events, such as suicidal ideation or behavior, violence and aggression to others, increase in depressive symptoms or anxiety, severe intoxication, or withdrawal symptoms, will all be managed with a crisis intervention delivered in the health center in accordance with local protocols. Close coordination between the study therapists and the rest of the health team will be encouraged through all protocol execution so that crisis protocols can be activated when necessary.

The study therapists will be responsible for assessing the participant when an adverse event is suspected and, if confirmed, will contact the trial coordinator and the local organization to mobilize the required resources (e.g., medical assessment, referral to the emergency room, and contact to a family member). The participants will have access to a centralized phone number where they can contact their therapist for help in case of emergency issues regarding treatment.

The trial coordinator will complete a report with the details of the event, including the diagnosis, management, and harms to the participant.

### Frequency and plans for auditing trial conduct {23}

There are two main areas where periodic audit procedures will take place: the clinical interaction among clients and therapists, and data integrity. All the therapy sessions will be audio-recorded, and then 10% of those will be randomly analyzed to check intervention fidelity. Also, by the end of each session, the client will report on the therapeutic alliance using a validated tool. Both procedures are meant to control the quality of the interventions being provided. Regarding data integrity, a research assistant with training in data supervision will verify the consistency and completeness of data each month.

### Plans for communicating important protocol amendments to relevant parties (e.g., trial participants, ethical committees) {25}

Any significant change to the protocol will require a formal amendment presentation to the ethics committee of the Pontificia Universidad Católica de Chile. Such amendments include, among others, changes to the primary outcome, sample size calculations, eligibility criteria, methods for blinding and allocation concealment, and statistical analysis.

### Dissemination plans {31a}

This trial originated from the collaborative work between our research team and the National Agency for Alcohol and Drugs (SENDA, its Spanish acronym). Additionally, the local primary care health services are providing critical support to the study. This proximity between our research team and the stakeholders should make dissemination easy, especially for the local context. In this regard, we will hold regular meetings with local stakeholders throughout the study and at least one meeting with SENDA to share the results at the end of the study.

Furthermore, as the results may be of interest to other PC services that address alcohol use disorders, we will publish the results in an international journal. The goal is to have the main manuscript submitted within 1 year from trial closure.

## Discussion

The present study will provide initial evidence on the effect of a brief psychosocial treatment for AUD in PC, which is essential in the context of the improved response capacity to harmful alcohol use through the SBIRT programs. The evidence on the effectiveness of such therapies in PC is still scarce, and this trial attempts to provide valuable information on the topic.

Based on previous reports [3, 4, 13], we hypothesized that the BMT would induce at least a 26% reduction of alcohol consumption compared to EUC. We think this is a reasonable superiority margin, over which the BMT could be further considered for incorporation into PC in Chile. The hypothesis will be tested by the analysis of the primary outcome, i.e., the change in the pattern of alcohol use 6 months after recruitment. We are aware that a binary response outcome would have been of more clinical significance; however, this test would have required a larger sample size, so it did not seem appropriate for an exploratory trial.

Some important modifying and predictive variables have been included to explain the results. The severity of the AUD is of cardinal importance since we do not expect BMT to be equally effective in all the spectrum of the disorder. Even though severity did not mediate MET effectiveness in the MATCH project [38], we have to bear in mind that the MATCH study occurred in specialized care settings and not in PC. In addition, PC is supposed to provide care for prevalent disorders in their mild to moderate presentations, so the question about the severity is of high relevance. Other essential modifiers are the fidelity to the intervention and the therapeutic alliance since both have shown to predict the effect of psychosocial interventions [39].

We devised BMT as a psychosocial intervention based on the best evidence available for effectiveness. Its procedures and strategies are an adaptation of the MET, a manualized therapy of well-proven effectiveness. Even though these procedures have been described in detail [19], it remains a reasonable question whether a general psychologist in PC will be able to provide the therapy with sufficient proficiency. Also, we assume that the participants receiving the BMT will be able to access additional services necessary for their recovery, for instance social services, physician consultations, among others. The study will provide some insights into how these factors mediate treatment effects on PC. Furthermore, the trial will describe to what extent the BMT adds therapeutic benefit to current care in PC.

In conclusion, this trial was conceived with a pragmatic approach, aiming to inform policymakers about the benefit of including a brief psychosocial treatment into PC.

## Trial status

Protocol version 1. April 10, 2020. Recruitment planned to start in September 2020 and last until September 2022.

## Supplementary information

**Additional file 1.**

## Abbreviations

ASI: Addiction Severity Index
AUD: Alcohol use disorder
AUDIT: Alcohol use disorders identification test
BMT: Brief motivational therapy
DALYs: Disability-adjusted life years
DDD: Drinks per drinking day
EUC: Enhanced usual care
FONDECYT: Fondo Nacional de Desarrollo Científico y Tecnológico
SDSS: Substance Dependence Severity Scale
DrInC: Drinker Inventory of Consequences
DSM-V: Diagnostic and Statistical Manual of Mental Disorders
MATCH: Matching Alcoholism Treatment to Client Heterogeneity
MD: Mean difference
MET: Motivational enhancement therapy
PC: Primary care
RA: Research assistant
RR: Relative risk
RRR: Relative risk reduction
SBIRT: Screening, brief intervention, and referral to treatment
SD: Standard deviation
SENDA: National service on drugs and alcohol (from the Spanish Servicio Nacional de Drogas y Alcohol)
TLFB: Timeline Follow Back
UKATT: United Kingdom Alcohol Treatment Trial
WAI—C/T: Working Alliance Inventory—client/therapist
