# Supplementary material for: Brief motivational therapy versus enhanced usual care for alcohol use disorders in primary care in Chile: study protocol for an exploratory randomized trial
Source: Trials. 2020 Jul 31;21:692. doi: 10.1186/s13063-020-04589-4 (PMC7393703; doi:10.1186/s13063-020-04589-4)
Supplement: Supplementary file 1 — Additional file 1. [file 13063_2020_4589_MOESM1_ESM.pdf]

INFORMED CONSENT DOCUMENT

(translated from the original in Spanish, see below)

Study Name: Brief motivational therapy versus enhanced usual care for alcohol use disorders in primary care in Chile

Funding Source: FONDECYT

Principal Investigator: Nicolás Barticevic Lantadilla

Contact phone: + 56 9 62225043

UC Department of Family Medicine

The purpose of this information is to help you decide to participate in this health - research project.

Take the time you need to decide yourself, read this document carefully, and ask the investigator-in-charge or study staff any questions you have.

RESEARCH OBJECTIVES

You have been invited to participate because your consumption of alcohol is causing some problems, or because you are seeking help with your drinking.

This study aims to find the best way to help people having problems with alcohol.

In total, we expect to have 182 participants in this study.

INVESTIGATION PROCEDURES

If you agree to participate in the study, you will be asked for the following:

· To complete a questionnaire about your health. Also, to answer questions about your drinking and its consequences.
· Then you will be asked to come back to this centre on two occasions and answer these same questions on the use of alcohol.
· These procedures do not alter the treatment you get to for your alcohol problem.

BENEFITS

You could benefit from participating in this study because you will receive appropriate help, which will be provided by experienced and trained professionals. In addition, the information that will be obtained will be useful to learn more about the problematic use of alcohol and could eventually benefit other people with the same condition.

RISKS

Participating in this study may cause you some type of discomfort because some of the questions may touch on sensitive or painful topics. You will be able to respond as far as you feel comfortable, without being forced to continue. In the event that you require professional support, as a result of the topics discussed, the team will assist you and / or to promptly refer you to the corresponding professional.

COSTS

Your participation in this study will not have any costs for you.

DAMAGE COVERAGE

Not considered.

COMPENSATIONS

You will be compensated for participating in this study by receiving a transportation card to attend the interviews of the study.

CONFIDENTIALITY OF INFORMATION

The information obtained will be kept confidential.
Your name, and any personal information will be kept in an encrypted database, which will only be accessed by professionals from the research team. In addition, at the end of the study, all the data that allows its identification will be erased from the records (name, RUN, etc).
This information will be stored indefinitely under the responsibility of the Responsible Investigator Dr. Nicolás Barticevic.
It is possible that the results obtained will be presented in magazines and medical conferences, however, your name will not be known.

WILLFULNESS

Your participation in this research is completely voluntary.
You have the right not to accept to participate or to withdraw your consent and to stop participating in this research at the time you deem appropriate. By doing so, you do not lose any rights that assist you as a patient of this institution, and the quality of medical care that you deserve will not be affected.

QUESTIONS

If you have questions about this research, you can contact or call
Dr. Nicolás Barticevic, Principal Investigator of the study.
Phone : +56 2 354 8584 and e mail: nabartic@uc.cl .

If you have questions about your rights as a participant in a medical research, you can call to Dr. Claudia Uribe Torres, Chairman of the 'Comité Ético Científico de la Facultad de Medicina de la Pontificia Universidad Católica de Chile'. Phone 223548173 , e-mail to : cecmeduc@med.puc.cl .

DECLARATION OF CONSENT

The purpose of this research, the procedures, the risks, the benefits and the rights that assist me and that I can withdraw from it at any time I wish, have been explained to me.

- I sign this document voluntarily, without being forced to do so.
- I am not waiving any rights.
- I will be informed of any new information related to the study that arises during this study and that may have direct importance for my health condition.
- I have been informed that I have the right to re - evaluate my participation in this research anytime I want to.
- I authorize the responsible researcher and his collaborators to access and use the data contained in my clinical file for the purposes of this investigation.
- At the time of signing, I am given a signed copy of this document.

MANDATORY SIGNATURES:

- Participant :

  Name : .... Signature .... Date................. ..

- Responsible Investigator :

  Name : .... Signature ………… .. ……… .. Date ……………….

- Director of the Institution or its Delegate:

  Name .... Signature .... Date................. ..

DOCUMENTO DE CONSENTIMIENTO INFORMADO

Nombre del Estudio: Efectividad de la terapia de activación motivacional para pacientes con trastorno por uso de alcohol en atención primaria en Chile.

Fuente Financiamiento: FONDECYT

Investigador Responsable: Nicolás Barticevic Lantadilla

Teléfono de contacto: + 56 9 62225043

Departamento de Medicina Familiar UC

El propósito de esta información es ayudarle a tomar la decisión de participar en una investigación en salud.

Tome el tiempo que necesite para decidirse, lea cuidadosamente este documento y hágale las preguntas que desee al investigador responsable o al personal del estudio.

OBJETIVOS DE LA INVESTIGACIÓN

Usted ha sido invitado / invitada a participar porque actualmente tiene un consumo de alcohol que le está trayendo problemas y está buscando ayuda para este problema.

El propósito de este estudio es buscar la manera más adecuada para proporcionar ayuda a las personas que están teniendo problemas con el consumo de alcohol.

En total, esperamos contar con 182 personas que participen en este estudio.

PROCEDIMIENTOS DE LA INVESTIGACIÓN

Si usted acepta participar en el estudio, se le solicitará lo siguiente:

- Completar un cuestionario de antecedentes personales, y responder algunas preguntas sobre el uso de alcohol en el último tiempo y algunas consecuencias que éste ha tenido para usted.
- Luego será contactado / contactada en dos ocasiones para que venga a este centro de salud; en estas oportunidades se le pedirá contestar nuevamente estos mismos cuestionarios sobre el uso de alcohol.
- Estos procedimientos no alteran el tratamiento que usted recibirá para el consumo de alcohol.

PONTIFICIA UNIVERSIDAD CATOLICA DE CHILE
COMITE ETICO CIENTIFICO
CEC MED UC
APROBADO
FACULTAD DE MEDICINA
10 / 10 / 2019

Beneficios

Usted podría verse beneficiado al participar de este estudio porque recibirá una ayuda apropiada, la que será brindada por profesionales con experiencia y formación.
Además, la información que se obtendrá será de utilidad para conocer más acerca del uso problemático de alcohol y eventualmente podría beneficiar a otras personas con su misma condición.

Riesgos

Participar en este estudio, le puede causar algún tipo de malestar o incomodidad porque algunas de las preguntas pueden tocar temas sensibles o dolorosos. Usted podrá responder hasta donde se sienta cómodo, sin ser forzado a continuar. En caso de que requiera contención o apoyo profesional, producto de los temas tratados, el equipo se compromete a asistirlo y/o derivar oportunamente al profesional correspondiente, en caso necesario.

Costos

Su participación en este estudio no le significara ningún costo para usted.

Cobertura de daños

No se considera cobertura de daños

Compensaciones

Usted será compensado por participar en este estudio recibiendo una tarjeta BIP para el pago del transporte para asistir a las entrevistas del estudio.

Confidencialidad de la información

La información obtenida se mantendrá en forma confidencial.
Su nombre, RUN, y cualquier información personal, serán mantenidos en una base de datos codificada, a la que sólo tendrán acceso los profesionales del equipo de investigación. Además, al finalizar el estudio, todos los datos que permitan su identificación serán borrados de los registros (nombre, RUN, etc).
Esta información será almacenada por tiempo indefinido bajo la responsabilidad del Investigador Responsable Dr. Nicolás Barticevic.
Es posible que los resultados obtenidos sean presentados en revistas y conferencias médicas, sin embargo, su nombre no será conocido.

PONTIFICIA UNIVERSIDAD CATOLICA DE CHILE
COMITE ETICO CIENTIFICO
CEC MED UC
APROBADO
FACULTAD DE MEDICINA
10/10/2019

Timbre, firma y fecha de aprobación

VOLUNTARIEDAD

Su participación en esta investigación es completamente voluntaria.
Usted tiene el derecho a no aceptar participar o a retirar su consentimiento y dejar de participar de esta investigación en el momento que lo estime conveniente. Al hacerlo, usted no pierde ningún derecho que le asiste como paciente de esta institución y no se verá afectada la calidad de la atención médica que merece.

PREGUNTAS

Si tiene preguntas acerca de esta investigación en salud puede contactar o llamar al Dr Nicolás Barticevic, Investigador Responsable del estudio, al teléfono: +56 23548584 y email: nbarticevic@med.puc.cl.

Si tiene preguntas acerca de sus derechos como participante en una investigación médica, usted puede llamar a la Dra. Claudia Uribe Torres., Presidente del Comité Etico Cientifico de la Facultad de Medicina, Pontificia Universidad Católica de Chile, al teléfono 223548173, o enviar un correo electrónico a: cecmeduc@med.puc.cl.

PONTIFICIA UNIVERSIDAD CATÓLICA DE CHILE
COMITE ETICO CIENTIFICO
CEC MED UC
APROBADO
FACULTAD DE MEDICINA
10/10/2019

Timbre, firma y fecha de aprobación

DECLARACIÓN DE CONSENTIMIENTO

Se me ha explicado el propósito de esta investigación, los procedimientos, los riesgos, los beneficios y los derechos que me asisten y que me puedo retirar de ella en el momento que lo desee.

- Firmo este documento voluntariamente, sin ser forzado/forzada a hacerlo.
- No estoy renunciando a ningún derecho que me asista.
- Se me comunicará de toda nueva información relacionada con el estudio que surja durante éste y que pueda tener importancia directa para mi condición de salud.
- Se me ha informado que tengo el derecho a reevaluar mi participación en esta investigación según mi parecer y en cualquier momento que lo desee.
- Yo autorizo al investigador responsable y sus colaboradores a acceder y usar los datos contenidos en mi ficha clínica para los propósitos de esta investigación.
- Al momento de la firma, se me entrega una copia firmada de este documento.

FIRMAS OBLIGATORIAS:

- Participante:

Nombre: ……………………… Firma…………………… Fecha………………..

- Investigador Responsable:

Nombre:……………………… Firma……………..……….. Fecha………………

- Director de la Institución o su Delegado:

Nombre……………………... Firma………………… Fecha………………..

PONTIFICIA UNIVERSIDAD CATOLICA DE CHILE COMITE ETICO CIENTIFICO CEC MED UC APROBADO FACULTAD DE MEDICINA 10/10/2019

Timbre, firma y fecha de aprobación
